# Youth in transition: Study protocol of a prospective cohort study into the long-term course of addiction, mental health problems and social functioning in youth entering addiction treatment

**DOI:** 10.1186/s12888-021-03520-8

**Published:** 2021-12-04

**Authors:** Christina Moska, Anna E. Goudriaan, Peter Blanken, Dike van de Mheen, Renske Spijkerman, Arnt Schellekens, Jannet de Jonge, Floris Bary, Wilma Vollebergh, Vincent Hendriks

**Affiliations:** 1 Parnassia Addiction Research Centre (PARC, Brijder Addiction Treatment), Zoutkeetsingel 40, 2512 HN The Hague, the Netherlands; 2 Department of Child and Adolescent Psychiatry, LUMC Curium, Leiden University Medical Center, Leiden, the Netherlands; 3 Department of Psychiatry, Academic Medical Centre, University of Amsterdam, Amsterdam, the Netherlands; 4 Amsterdam Institute for Addiction Research, Arkin Mental Health Care, Amsterdam, the Netherlands; 5 Department of Tranzo Scientific Center for Care and Wellbeing, Tilburg University, Tilburg, the Netherlands; 6 Department of Psychiatry, Radboud University Medical Centre, Donders Institute for Brain, Cognition, and Behavior, Nijmegen, the Netherlands; 7 Nijmegen Institute for Science Practitioners in Addiction (NISPA), Nijmegen, the Netherlands; 8 Research Group Urban Vitality, Faculty of Health, Amsterdam University of Applied Science, Amsterdam, the Netherlands; 9 Netherlands Network of Client Councils in Addiction Care ‘Het Zwarte Gat’, Hollands Kroon, The Netherlands; 10 Department of Interdisciplinary Social Science, Utrecht University, Utrecht, the Netherlands

**Keywords:** Adolescents, Youth addiction treatment, Substance use disorder, Long-term course of SUD, Prospective cohort study

## Abstract

**Background:**

Substance use disorders (SUDs) are prevalent in the general population, tend to follow a chronic course, are associated with many individual and social problems, and often have their onset in adolescence. However, the knowledge base from prospective population surveys and treatment-outcome studies on the course of SUD in adolescents is limited at best. The present study aims to fill this gap and focuses on a subgroup that is particularly at risk for chronicity: adolescents in addiction treatment. We will investigate the rate of persistent SUD and its predictors longitudinally from adolescence to young adulthood among youth with DSM-5 SUD from the start of their addiction treatment to 2 and 4 years following treatment-entry. In addition to SUD, we will investigate the course of comorbid mental disorders, social functioning, and quality of life and their association with SUD over time.

**Methods/design:**

In a naturalistic, multi-center prospective cohort design, we will include youths (*n* = 420), who consecutively enter addiction treatment at ten participating organizations in the Netherlands. Inclusion is prestratified by treatment organization, to ensure a nationally representative sample. Eligible youths are 16 to 22 years old and seek help for a primary DSM-5 cannabis, alcohol, cocaine or amphetamine use disorder. Assessments focus on lifetime and current substance use and SUD, non-SUD mental disorders, family history, life events, social functioning, treatment history, quality of life, chronic stress indicators (hair cortisol) and neuropsychological tests (computerized executive function tasks) and are conducted at baseline, end of treatment, and 2 and 4 years post-baseline. Baseline data and treatment data (type, intensity, duration) will be used to predict outcome – persistence of or desistance from SUD.

**Discussion:**

There are remarkably few prospective studies worldwide that investigated the course of SUD in adolescents in addiction treatment for longer than 1 year. We are confident that the Youth in Transition study will further our understanding of determinants and consequences of persistent SUD among high-risk adolescents during the critical transition from adolescence to young adulthood.

**Trial registration:**

The Netherlands National Trial Register Trial NL7928. Date of registration January 17, 2019.

## Background

Substance use disorders (SUDs) are prevalent in the general adult population [1], tend to follow a chronic or chronic-intermittent course, are associated with many individual and social problems, and – as most mental disorders – often have their onset in adolescence [2]. Hence, the course of SUD among adolescents and young adults should be high on our research agenda. Nevertheless, the literature regarding the development, persistence or desistance and treatment of SUD in youth is sparse, and research in this area lags considerably behind that of research in adult SUD.

When studying substance use ‘trajectories’ several transitions can be distinguished. The first series of transitions cover the initiation of substance use, and the transition from first use to regular or frequent use, and from frequent use to disordered use. The subsequent transition pertains to the persistence or desistance of SUD. In the present study we examine the last transition – the persistence of or desistance from SUD in the period from late adolescence (i.e., 16 to 22 yrs) to early adulthood (20 to 26 yrs) – among youth in addiction treatment.

Various prospective studies have traced the progression from first substance use through frequent consumption to the onset of SUD in community samples of adolescents, with most studies pertaining to cannabis, the most commonly used illicit drug worldwide [3–7]. Most substance use in adolescents remains experimental, irregular or modest. However, it tends to become more frequent, intensive and long-lasting when the use of substances is part of multiple problem behavior including comorbidity with mental health problems, social dysfunction, and delinquency [7–9].

Much less is known about what happens next: the course of SUD during the critical transition period from adolescence to adulthood. Based on Moffitt’s taxonomy of antisocial behavior [10, 11], the literature distinguishes between adolescent-limited and life-course-persistent trajectories of substance use and SUD, but few prospective population studies have in fact addressed this issue, and virtually all studies were conducted in the United States, Australia and New Zealand [12–16]. For example, Meier et al. (2015) investigated the development of persistent substance dependence on alcohol, nicotine, cannabis or hard drugs from adolescence to adulthood up to 38 years in the Dunedin birth cohort in New Zealand, and found a persistence rate of substance dependence of 19% when including nicotine dependence, and of 9% when excluding nicotine dependence from the outcome. In addition, they found a specific set of childhood and adolescent risk factors, including family history of SUD, childhood psychopathology, and childhood socioeconomic status, to be strongly associated with persistent substance dependence in adulthood. Only a few European population studies investigated the course of SUD from adolescence to young adulthood, but these focused on cannabis only [17, 18].

The present study focuses on a population that is particularly at risk for chronicity: adolescents in addiction treatment. Although research on treatment outcome of adolescents with SUD has expanded in the past decade [19], there are remarkably few studies that investigated the course of SUD in adolescents following addiction treatment prospectively for longer than 1 year. We found only three large-scale (*N* > 300) studies that did so [20–22]. Results from these – all US-based – studies suggest that, despite overall treatment benefit, roughly 15 to 30% of treated adolescents show a pattern of persistent SUD/dependence or even deterioration during the 3 to 6 years post-treatment.

To conclude, the available data from prospective population surveys and treatment-outcome studies on the course of SUD in adolescents are limited at best. Remission, continuation and progression rates are largely unknown, as are the risk and protective factors involved. While nearly 6000 youth aged 22 years or younger enter treatment for SUD annually in the Netherlands [23], we have virtually no information as to how these youth will fare moving from adolescence to early adulthood. The present study aims to fill these gaps by addressing the study objectives outlined below.

## Study objectives

In this study, we will investigate the rate of persistent SUD and its predictors longitudinally from adolescence to young adulthood in a representative treatment sample of youth with DSM-5 SUD from the start of their addiction treatment to 2 and 4 years following treatment-entry. In addition to SUD, we will investigate the course of comorbid mental disorders and social functioning, and their relation with SUD over time.

Our primary study questions are:

1. What is the persistence rate of DSM-5 moderate to severe SUD from adolescence to young adulthood among youth in the two and four years following entry in addiction treatment?
2. What is the prognostic value of a general population-based set of predictors of persistent SUD – based on the Dutch Tracking Adolescents’ Individual Lives Survey (TRAILS) study – from adolescence to young adulthood for predicting persistent DSM-5 moderate to severe SUD among youth in addiction treatment?
3. Can we optimize the accuracy of predicting persistent DSM-5 moderate to severe SUD among youth in addiction treatment by extending or modifying the general population-based set of predictors with baseline indicators from our addiction treatment sample?
4. Which longitudinal treatment outcome trajectories can be identified from adolescence to young adulthood pertaining to substance use and SUD, comorbid mental health problems, and social functioning among youth in addiction treatment?
5. Which treatment interventions – in terms of type, intensity and duration – are associated with favorable or unfavorable long-term outcomes, in terms of substance use and SUD, mental health problems and social functioning, for which youth in addiction treatment?

## Methods

### Research design

Our study is designed as a naturalistic, multi-center prospective cohort study among youth seeking help in addiction treatment to investigate the study objectives mentioned above.

We will recruit youth aged 16 to 22 years (*N* = 420) who enter addiction treatment at ten participating treatment organizations from a total of 13 organizations in the Netherlands. Participants will be assessed on a range of measures related to substance use and SUD, comorbid mental health problems and social functioning at treatment-entry (baseline), end of treatment, and 2 and 4 years post-baseline. Study-inflow of participants consists of consecutive admissions, and will be prestratified by treatment organization to ensure a nationally representative sample. Treatment consists of treatment as usual, as provided by the participating addiction care organizations, to maximize generalizability of the study findings to everyday clinical practice. Analyses will be conducted following an intention-to-treat approach.

This study was funded by The Netherlands Organization for Health Research and Development (60–63,600–98-317) and was approved by the Medical Ethical Board of the Leiden University Medical Center (NL65903.058.18; file number P18.175). Trial registration: NL7928.

### Participants and setting

**Fig. 1:**
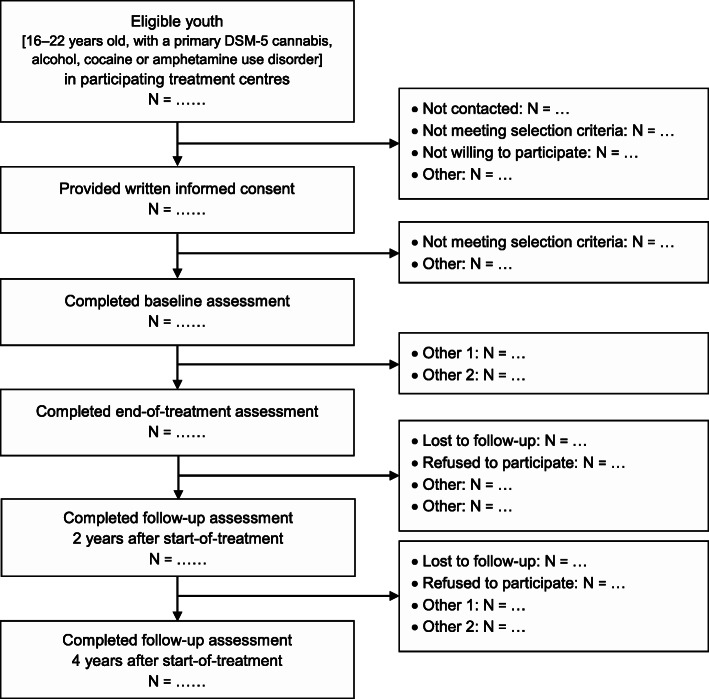
Flowchart of the Youth in Transition study

Based on national treatment registration data [23], a total of 5769 youth up to age 22 years applied for addiction treatment in the Netherlands in 2015. From these, the far majority (*N* = 5151; 89%) were 16 to 22 years old, and most sought help for a primary cannabis- (53%), alcohol- (17%) or stimulant- (11%, cocaine or amphetamine) related problem. Hence, 81% (*N* = 4169) of total treatment demand among youth aged 16–22 years concerns cannabis, alcohol, cocaine and/or amphetamines, with other substances (e.g., opiates, ecstasy, GHB, and benzodiazepines) each representing less than 2% of treatment demand. Limiting our inclusion to youth aged 16–22 years, with the primary substances mentioned above, and using a sampling fraction of 10%, 420 youth will be included in our study (see Fig. 1).

Inclusion criteria are: age 16 to 22 years; primary SUD pertains to cannabis, alcohol, cocaine or amphetamines; willingness to participate in the study (oral and written informed consent). Exclusion criteria are: referral for diagnostic evaluation only; insufficient comprehension of basic Dutch language. The ten participating organizations include specialized addiction treatments and integrated mental health and addiction treatments, with treatment covered by the health insurance system. Recruitment of study participants takes place at 41 treatment locations situated throughout the Netherlands.

### Treatment

Treatment consists of the usual treatment-offer provided by the addiction care organizations to maximize ecological validity of the study outcomes. We will document the type of treatment received for SUD, which in the Netherlands mostly consists of integrated cognitive behavioral therapy plus motivational interviewing (CBT + MI), community reinforcement approach (CRA), family therapy, and CBT-based e-health interventions. In addition, we will document – psychosocial and/or pharmacological – treatment for comorbid mental health problems as well as intensity (inpatient; outpatient) and actual duration (retention) of treatment, and type of treatment termination (premature/planned).

### Assessments

Study assessments take place at study-entry (baseline), end of treatment, and 2 and 4 years post-baseline, and will be conducted by trained, academic level, research assistants, who are independent from the treatment-staff. Youth who apply for treatment at the participating treatment centers will be approached by treatment-staff with the request to participate in the study. Following screening for eligibility, participants are asked to provide informed consent, after which the baseline assessment and subsequent two- and four-years follow-up will be conducted.

For the purpose of the present study, we developed the Youth in Transition Questionnaire (YIT-Q [24]), which contains both widely used instruments like the Depression, Anxiety and Stress Scale (DASS-21 [25]) and the World Health Organization Disability Assessment Schedule (WHODAS-2.0 [26]) as well as domains with item-sets that were developed by our research group (see Table 1). Both the DASS-21 and the WHODAS-2.0 have shown a stable factor structure, and their scales have excellent internal consistency reliability and good convergent en discriminant validity (e.g., [27, 28]).

**Table 1:** Assessment domains and instruments

	Instrument	Baseline	End of treatment	2 year FU	4 year FU
**Demographic background**					
• Age; sex; cultural background/identity	YIT-Q	X			
• Family educational level and socioeconomic status	YIT-Q	X			
**Substance use**					
• Lifetime and recent substance use	MATE-Y	X		X	X
• DSM-5 substance use disorder (SUD); age of onset	MATE-Y	X		X	X
**Non-SUD mental disorders**					
• DSM-5 lifetime and past-year mental disorders; age of onset	Structured DSM-5 interview	X		X	X
• Dimensional measures of mental health problems, including depression, anxiety, stress; prodromal psychotic symptoms; personality risk for substance abuse; intellectual disability	DASS-21; PQ-16;	X		X	X
	SURPS; SCIL	X			
• Suicidal ideation/attempts	MINI			X	X
**Family history/environment**					
• History of SUD (parents; grandparents; siblings)	YIT-Q	X			
• History of non-SUD mental disorders; criminality, violence, abuse, neglect, homelessness	YIT-Q	X			
	YIT-Q	X			
**Life events**					
• Traumatic life events	Structured DSM-5 interview	X		X	X
• Turning points with major impact on addiction course	YIT-Q			X	X
**Social functioning**					
• Youth’s functioning in the areas of: education; employment; living arrangements; social relationships/support; delinquency	YIT-Q	X		X	X
• General functioning, disability and quality of life	WHODAS-2.0	X		X	X
**Treatment history**					
• Number/types of earlier addiction and mental health treatments	YIT-Q	X		X	X
• Intention/motivation for current treatment	YIT-Q	X			
**Endophenotype and biomarker**					
• Behavioral measures of impulsivity (computerized tasks)	Risky Gains task, VBDM, Self-Ordered Pointing task	X		X	X
• Biomarkers of chronic stress: hair cortisol and testosterone	Hair sample analysis	X		X	X
**Index treatment characteristics**					
• Type, intensity, duration of current treatment received and initial treatment response^a^			X		

*DASS-21* Depression, Anxiety and Stress Scale, *MATE-Y* Measurements in the Addictions for Triage and Evaluation – Youth version, *PQ-16* Prodromal Questionnaire – 16 item version, *SCIL* Screener for Intelligence and Learning disability, *SURPS* Substance Use Risk Profile Scale, *VBDM* Valued-based decision-making battery, *WHODAS-2.0* World Health Organization Disability Assessment Schedule, *YIT-*Q Youth In Transition Questionnaire

^a^Note: All information at the end of treatment assessment will be obtained from the responsible clinician

At baseline, we collect sociodemographic information and administer the substance use section of the Measurements in the Addictions for Triage and Evaluation – Youth version (MATE-Y [29]), which is the standard intake instrument in most youth addiction treatment organizations in the Netherlands. Both lifetime (onset, duration) and past-month (days of use) substance use data are collected, and the criteria of the fifth edition of the Diagnostic and Statistical Manual of Mental Disorders (DSM-5 [30]) are administered to obtain a lifetime and past-year diagnosis of SUD for the primary and – if present – secondary substance for which the youths seek help. To assess the presence of comorbid non-SUD mental disorders, we developed a structured DSM-5 diagnostic interview which covers ten of the most common disorders: depression; panic disorder; agoraphobia; social phobia; post-traumatic stress disorder; attention-deficit/hyperactivity disorder; conduct disorder; anorexia nervosa and bulimia nervosa; generalized anxiety disorder. For each disorder, the main diagnostic criteria are used as screening questions, and only if these main criteria are met in a period that the person was not intoxicated and did not experience alcohol or drug withdrawal symptoms – hence, do not reflect a substance-induced disorder only – the full section of that disorder is administered. In addition to DSM-5 classification, the DASS-21 (depression, anxiety, stress), Prodromal Questionnaire (PQ-16 [31]) (subclinical psychotic symptoms), Substance Use Risk Profile Scale (SURPS [32]) (personality risk for substance abuse) and Screener for Intelligence and Learning disability (SCIL [33]) are administered to obtain dimensional measures of mental health, and the WHODAS-2.0 is used to assess general functioning, disability and quality of life. The PQ-16 was found to accurately identify ultra high risk (UHR) and full psychosis, with area under the curve (AUC) values ranging from 0.71 to 0.95 [34], and the SURPS showed adequate to high internal consistency (Cronbach’s alpha = 0.61–0.86), a stable factor structure that reflects the four scales of the instrument, and good test-retest reliability, with intra-class correlation coefficients (ICC) ranging from 0.68 to 0.88 [35]. The SCIL has shown excellent test-retest reliability (Pearson’s r = 0.92) and good predictive validity for detecting mild or borderline intellectual disabilities in both adults (AUC = 0.93) and youths (AUC = 0.91) [33].

The YIT-Q assessment domains pertaining to family history/environment, social functioning and treatment history/motivation all contain structured questions which inform about the youth’s self-reported perception of his/her own functioning or problems in these areas, or – in the case of family history – his/her perception of his family members’ functioning/problems (see Table 1). Questions pertaining to suicidal ideation/attempts were derived from the Mini International Neuropsychiatric Interview (MINI, version 5.0.0 [36]). For assessing family history of SUD, non-SUD mental disorders and criminality we used the Family History Method (FHM [37]) as described by Schuckit et al. (2020). Given that the FHM tends to underestimate family diagnosis, we aimed to increase its sensitivity by asking the youth whether his/her (grand)parent(s) or sibling(s) had ever received treatment for alcohol, drug or mental health problems, or whether they had ever been incarcerated. We derived and adapted a set of questions about family history of violence, abuse and neglect towards the youth from the International Society for the Prevention of Child Abuse & Neglect (ISPCAN) Child Abuse Screening Tools (ICAST), version for young adults (ICAST-R [38]). For assessing important ‘turning points’ that had a major impact on the course of the youth’s substance use career, we will use a qualitative approach with open-ended questions [39]. Questions on the youth’s social functioning, including employment, living arrangements, social support and delinquency were derived and adapted from item-sets in the Substance Use Recovery Evaluator (SURE [40]) and the Treatment Outcomes Profile (TOP [41]). Both instruments are widely used and validated, and are included in the recent International Consortium for Health Outcomes Measurement (ICHOM [42]) standard set for addiction. For example, the five subscales of the SURE showed good internal consistency (alpha = 0.68–0.87), and reflect a stable factor structure of the instrument [40]. In addition, all items of the TOP in the areas of employment, living arrangements and delinquency showed ‘substantial’ or ‘good’ inter-rater reliability, with Cohen’s kappa> 0.61, and ICC > 0.75, respectively [41]. We limited our assessment of intention/motivation for treatment to a single-item question pertaining to the youth’s self-reported primary goal of treatment – total abstinence or strong/small/no reduction of substance use.

In addition to the self-reported measures described above, we administer several computerized tasks pertaining to impulsive or risky choice and value-based decision-making (VBDM). Specifically, we include a delay discounting task, in which four versions are administered: a) delay discounting; b) probability discounting of gains; c) probability discounting of losses; d) mixed gambles. The task is administered using MATLAB software; further details on task description can be found in [43]. In addition, a computerized self-ordered pointing task is administered using Inquisit software, based on Petrides and Milner (1982) from the Inquisit test library [44]. Lastly, we administer an Inquisit version of the Risky Gains Task developed by Paulus et al. (2003) [45, 46]. Concerning discounting in adolescents, studies have shown that individual differences in decision-making are stable over time, as indicatated by moderate to good test-retest reliability even over a 2-year interval, with ICC-values ranging from 0.65 to 0.78 [47]. Lastly, we collect a scalp hair sample of (if possible) at least 3 cm and analyze hair cortisol and testosterone to obtain a biological measure of chronic stress. Hair cortisol analysis is increasingly used in mental health research, and is a reliable method to determine cortisol levels over a prolonged period of time [48, 49]. In addition, it has recently been shown that psychosocial stress increases testosterone levels in both healthy controls and patients with posttraumatic stress-disorder and borderline personality disorder [50]. We therefore also assess hair testosterone levels, in order to have an additional read-out for physiological effects of chronic stress and explore a potential link between elevated testosterone and impulse regulation issues among youth with substance use disorders.

The end-of-treatment assessment will be limited to documenting the judgement of the youth’s primary clinician about the youth’s short-term treatment response (partial or full remission; persistence; deterioration of problematic substance use) and documenting the treatment received (type; intensity; duration) and type of treatment termination (premature; planned).

With the exception of most demographic background variables and questions pertaining to pre-baseline history, the two- and four-year follow-up interviews will focus on the same domains and include the same instruments as the baseline assessment, with time-frames adapted to the follow-up period. In addition, we will inquire whether the youth experienced important ‘turning points’ or major life events that, in his or her view, had a major impact on the course of his/her substance use in two- and four-years follow-up period.

Time to administer the baseline and follow-up assessments is estimated at 2 and 1.5 h each, respectively. The assessments will preferably be conducted at the treatment center. Youth will receive a remuneration of 20, 30 and 50 euro for participating in the baseline and two- and four-year follow-up, respectively. In addition, they have the possibility to win a gift certificate of 200 euro (6 certificates available) for follow-up attendance, as has been done successfully in previous studies [51].

### Power considerations

Using a sampling fraction of 10% to obtain a representative sample from the total target population (*N* = 4169; see ‘Participants and setting’ above), we aim to include 420 youth in our study. Concerning our first study question, we assume a 30% persistence rate of moderate to severe SUD in our study sample [20, 21, 52]. With precision at 5% [53], a minimum sample size of *N* = 300 would be sufficient to estimate the assumed 30% persistence rate with a 95% level of confidence [54].

However, for our prediction analyses of persistent SUD (study questions 2, 3 and 5) we need a larger sample size than N = 300 to allow a sufficiently large number of predictor variables to be included in the logistic regression models. To avoid overfit of a logistic regression model, authors recommend a minimum of 10 events (i.e., 10 patients with persistent SUD) for each predictor variable [55–57]. Hence, given the assumed 30% persistence rate, and *N* = 420, a maximum of 12 predictor variables could be included in the multivariate logistic regression analysis.

### Data analysis

Study data will be analyzed following an intent-to-treat (ITT) approach. The ITT-population consists of all patients who provide informed consent and receive at least one treatment session.

1. What is the persistence rate of DSM-5 moderate to severe SUD from adolescence to young adulthood among youth in the 2 and 4 years following entry in addiction treatment?

Persistent moderate to severe SUD is defined as meeting the criteria of lifetime moderate to severe SUD for alcohol, cannabis, cocaine or amphetamines – collapsed across substances – at baseline, as well as meeting the criteria of past-year moderate to severe SUD at both the 2 year and 4 year follow-up. According to DSM-5, moderate to severe SUD is defined as meeting four or more of the 11 SUD-criteria. In case of missing follow-up data we will conduct sensitivity analyses, using multiple imputation methods based on post-baseline treatment, end-of-treatment and follow-up data that we do have available of a participant. We will also conduct a ‘worst-case’ scenario analysis, in which those not reached at both the two- and four-year follow-up are considered as youth with persistent SUD. Notably, for youth without past-year SUD at the 2 year follow-up and a missing 4 year follow-up (and vice versa), our primary outcome variable is available: they are – by definition – youth with non-persistent SUD.

(2) What is the prognostic value of a general population-based set of predictors of persistent SUD from adolescence to young adulthood for predicting persistent DSM-5 moderate to severe SUD among youth in addiction treatment?

We will use existing data from the Tracking Adolescents’ Individual Lives Survey (TRAILS) study to investigate the rate of persistent substance dependence (DSM-IV) and its predictors from adolescence to young adulthood in the general population. TRAILS is a prospective cohort study (*N* = 2230) into the development of mental health from preadolescence (age 11 years) to adulthood (up to age 26 years) in the Dutch general population with five biennial or triennial follow-up waves [58]. Candidate-predictors stem from the areas of: demographic background; substance use and SUD; non-SUD mental disorders; family history; life events; and social functioning (see first six domains in Table 1). Logistic regression will be used to investigate the relation of these predictors with the outcome variable, persistent substance dependence, following the two-step procedure suggested by Hosmer and Lemeshow (2000) [59]. Only variables which are significantly associated with the outcome variable in the first, univariate, step will be entered as candidates into the multivariate (backward) logistic regression model in the second step. Next, we will evaluate the accuracy of the set of combined adolescent risk factors found to predict persistent substance dependence in the TRAILS cohort for predicting persistent DSM-5 moderate to severe SUD among youth in addiction treatment, in terms of sensitivity, specificity, and positive and negative predictive value.

(3) Can we optimize the accuracy of predicting persistent DSM-5 moderate to severe SUD among youth in addiction treatment by extending or modifying the general population-based set of predictors with baseline indicators from our addiction treatment sample?

This question will be addressed (a) by building a new prediction model based upon the same potential predictor variables as used in the TRAILS analysis above, and supplement these with potential predictor variables assessed at baseline in our addiction treatment sample (Table 1), and (b) by comparing the accuracy of this treatment-based prediction model with the TRAILS-population-based prediction model.

(4) Which longitudinal treatment outcome trajectories can be identified from adolescence to young adulthood pertaining to substance use and SUD, comorbid mental health problems, and social functioning among youth in addiction treatment?

Concerning the fourth study question, we will use the total count of DSM-5 SUD-symptoms derived from the MATE-Y, and the sum score on the DASS-21 (mental health problems) and WHODAS 2.0 (social functioning), assessed at baseline and at two- and four-years follow-up (Table 1). To describe the longitudinal course of SUD, mental health problems and social functioning we will employ latent growth curve modeling [60].

(5) Which treatment interventions – in terms of type, intensity and duration – are associated with favorable or unfavorable long-term outcomes, in terms of substance use and SUD, mental health problems and social functioning, for which youth in addiction treatment?

The fifth study question will be answered in two successive steps. First, we will determine which treatment interventions are associated with a favorable treatment outcome, using the same two-step logistic regression procedure as described for study question 3. Next, we will determine whether the youth-related potential predictors for persistent SUD (see study question 3) moderate the favorable or unfavorable long-term outcomes of the different treatment interventions. To determine which treatment interventions are associated with a favorable long-term – two- and four-years – outcome, we will categorize the treatment received along the following dimensions: type of treatment; intensity; duration; type of termination (see section ‘Treatment’ above).

All study questions will be analyzed using the statistical software packages of SPSS version 25, R, and Mplus, where appropriate.

## Discussion

This study aims to investigate the long-term course of SUD, concurrent mental health problems and social functioning in a group that is particularly at risk for chronicity: youth in addiction treatment. Strengths of the study are (1) obtaining a nationally representative sample of youth in addiction treatment, (2) the relatively large size of our cohort, (3) the use of both self-report and endophenotypical biological and cognitive-behavioral data, and (4) our aim to determine the long-term outcome of youth addiction treatment in a naturalistic, ‘real world’ treatment context. Study limitations include the following. First, our study sample consists of youth aged 16 to 22 years. We would have preferred to include younger adolescents as well, but Dutch national registration data showed that only 618 youths aged 15 years and younger seek help at addiction treatment annually (i.e., 11% of total youths’ treatment demand). Second, our maximum follow-up period is 4 years. We would have preferred a longer follow-up period (e.g., 8 to 10 years) to sufficiently cover the age of young adulthood, but this was not possible due to budget limitations. Third, our study data are in essence correlational in nature, and hence, are subject to confounding. Including a non-treatment control group would limit confounding, but for reasons of feasibility, ethics and costs, we chose not to include a non-treatment (matched) control cohort in our study. Another option would have been to conduct a large-scale randomized controlled trial (RCT), but for obvious reasons an RCT with a non-treatment control group would be unacceptable for ethical reasons. Nevertheless, we will include important and well-known confounders like social economic status in our analyses. Lastly, most of our data are based on self-report and may be subject to underreporting. Self-reported substance use can be verified by means of urinalyses, but this method only provides a momentary assessment and does not give an indication of amount or severity of use. Studies indicate that self-report is quite reliable if confidentiality is guaranteed and if no negative consequences are attached to the answers provided by the person [61, 62]. To assure that this will be the case, our study assessments will be conducted by research-assistants that are independent from treatment-staff. Nevertheless, youths with insufficient comprehension of basic Dutch language will be barred from the study (exclusion criterion), and hence this potentially important subpopulation will not be represented in our study data.

All in all, we are confident that this study will further our understanding of (1) the course of SUD in youths seeking help for their addiction problems, (2) the relationship between SUD and comorbid mental disorders and social functioning over time, and (3) the determinants and consequences of persistent SUD during the transition from adolescence to young adulthood.

## Data Availability

Not applicable, because our manuscript does not contain any data; it is a study protocol paper.

## Abbreviations

CBT: Cognitive behavioral therapy
CRA: Community reinforcement approach
DASS-21: Depression, anxiety and stress scale
DSM-IV: Diagnostic and statistical manual of mental disorders, fourth edition
DSM-5: Diagnostic and statistical manual of mental disorders, fifth edition
ITT: Intent-to-treat
MATE-Y: Measurements in the addictions for triage and evaluation – youth version
MI: Motivational interviewing
PQ-16: Prodromal questionnaire
RCT: Randomized controlled trial
SCIL: Screener for intelligence
SPSS: Statistical package for the social sciences
SUD: Substance use disorder
SURPS: Substance use risk profile scale
TRAILS: Tracking adolescents’ individual lives survey
YIT: Youth in transition
YIT-Q: Youth in transition questionnaire
WHODAS-2.0: World health organization disability assessment schedule.
